# Concomitant tricuspid regurgitation severity and its secondary reduction determine long-term prognosis after transcatheter mitral valve edge-to-edge repair

**DOI:** 10.1007/s00392-020-01798-4

**Published:** 2021-01-12

**Authors:** Martin Geyer, Karsten Keller, Kevin Bachmann, Sonja Born, Alexander R. Tamm, Tobias Friedrich Ruf, Felix Kreidel, Omar Hahad, Aniela Petrescu, Michaela Hell, Andres Beiras-Fernandez, Angela Kornberger, Eberhard Schulz, Thomas Münzel, Ralph Stephan von Bardeleben

**Affiliations:** 1 Department of Cardiology, Cardiology I, University Medical Center Mainz (Johannes Gutenberg-University Mainz), Mainz, Germany; 2 Center for Thrombosis and Hemostasis (CTH), University Medical Center Mainz (Johannes Gutenberg-University Mainz), Mainz, Germany; 3 Department of Sports Medicine, Medical Clinic VII, University Hospital Heidelberg, Heidelberg, Germany; 4 German Center for Cardiovascular Research (DZHK), Partner Site Rhine Main, Mainz, Germany; 5 Department for Cardiothoracic and Vascular Surgery, University Medical Center Mainz (Johannes Gutenberg-University Mainz), Langenbeckstr 1, 55131 Mainz, Germany

**Keywords:** Tricuspid regurgitation, Mitral regurgitation, Mitral valve repair, MitraClip, Multidisciplinary heart team, Survival

## Abstract

**Background:**

Concomitant tricuspid regurgitation (TR) is a common finding in mitral regurgitation (MR). Transcatheter repair (TMVR) is a favorable treatment option in patients at elevated surgical risk. To date, evidence on long-term prognosis and the prognostic impact of TR after TMVR is limited.

**Methods:**

Long-term survival data of patients undergoing isolated edge-to-edge repair from June 2010 to March 2018 (combinations with other forms of TMVR or tricuspid valve therapy excluded) were analyzed in a retrospective monocentric study. TR severity was categorized and the impact of TR on survival was analysed.

**Results:**

Overall, 606 patients [46.5% female, 56.4% functional MR (FMR)] were enrolled in this study. TR at baseline was categorized severe/medium/mild/no or trace in 23.2/34.3/36.3/6.3% of the cases. At 30-day follow-up, improvement of at least one TR-grade was documented in 34.9%. Severe TR at baseline was identified as predictor of 1-year survival [65.2% vs. 77.0%, *p* = 0.030; HR for death 1.68 (95% CI 1.12–2.54), *p* = 0.013] and in FMR-patients also regarding long-term prognosis [adjusted HR for long-term mortality 1.57 (95% CI 1.00–2.45), *p* = 0.049]. Missing post-interventional reduction of TR severity was predictive for poor prognosis, especially in the FMR-subgroup [1-year survival: 92.9% vs. 78.3%, *p* = 0.025; HR for death at 1-year follow-up 3.31 (95% CI 1.15–9.58), *p* = 0.027]. While BNP levels decreased in both subgroups, TR reduction was associated with improved symptomatic benefit (NYHA-class-reduction 78.6 vs. 65.9%, *p* = 0.021).

**Conclusion:**

In this large study, both, severe TR at baseline as well as missing secondary reduction were predictive for impaired long-term prognosis, especially in patients with FMR etiology. TR reduction was associated with increased symptomatic benefit.

**Graphic abstract:**

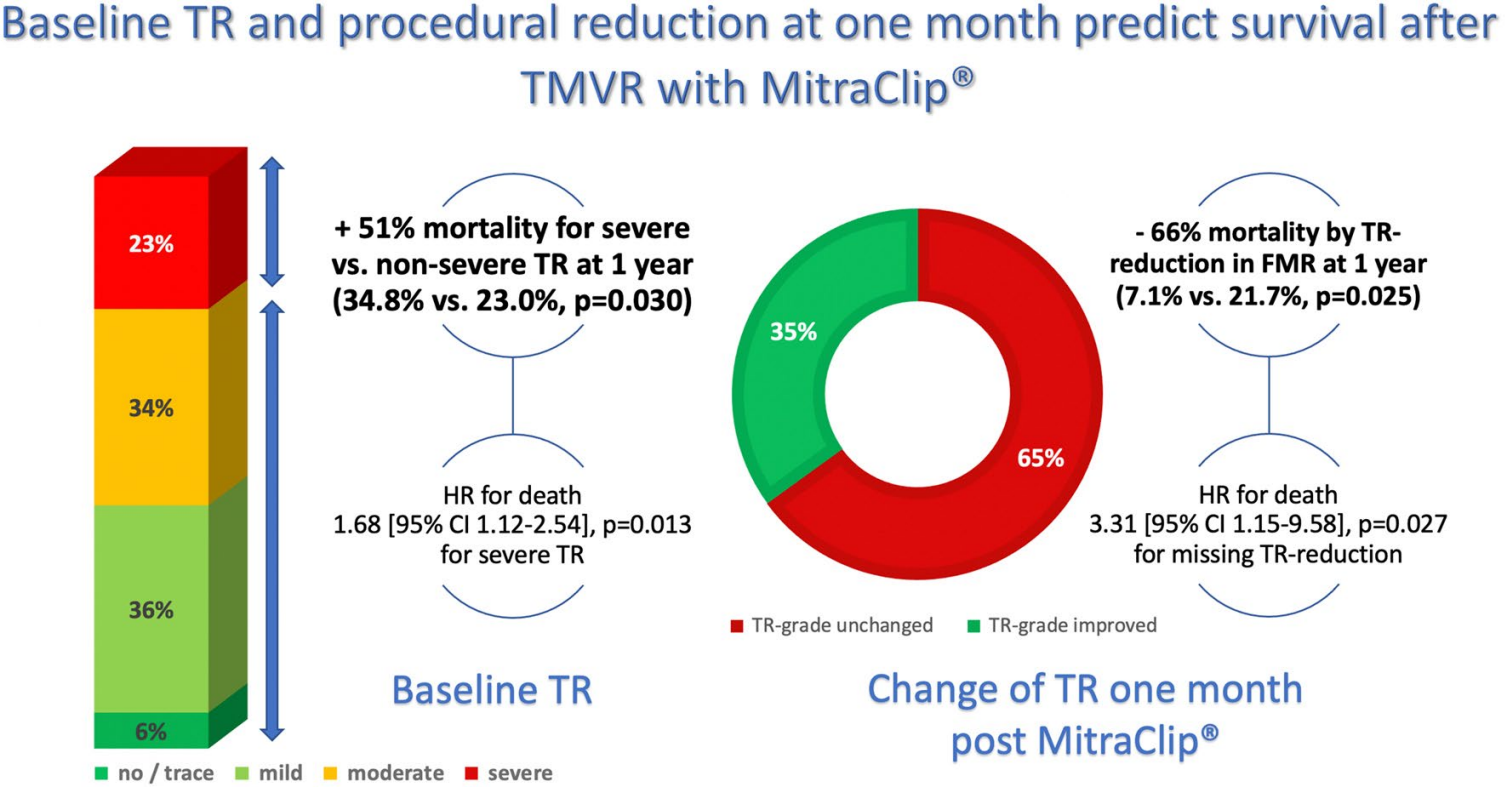

## Introduction

Mitral valve regurgitation (MR) is a common valvular disorder with an age-dependent prevalence exceeding 10% in individuals over 75 years [1]. As many of these patients are at relevantly elevated surgical risk [2], percutaneous minimally invasive transcatheter mitral valve repair (TMVR) has become a frequently used treatment. While several devices have been introduced, the so-called “edge-to-edge” therapy (e.g., MitraClip^®^, Abbott Vascular, Santa Clara, CA, USA) is the most established type of TMVR.

Tricuspid valve regurgitation (TR) is a common finding in elder patients: the Framingham Heart Study reported on an incidence of moderate or severe TR in 5.6% of female and 1.5% of male individuals aged 70 years and older [3]. Secondary TR constitutes the predominant pathomechanism with over 90% of the cases and is often found in the context of left ventricular heart disease. In this context, TR was shown to be significantly associated with the presence of MR and potentially related to MR severity [4]. Relevant TR can be detected in 30–50% of patients with MR [5, 6]. TR was shown to be an individual predictor for adverse prognosis in large registries [7] also in the context of MR [8]. Contemporarily, only a minority of TR patients are treated up to now [9]. There is evidence, that TMVR is capable of reducing severity of concomitant TR in approximately a fourth to a third of the MR patients [10, 11]. With the development of treatment devices dedicated to interventional tricuspid valve repair for patients at elevated surgical risk, the question of a prognostic impact of TR in patients with significant MR gains further relevance. Furthermore, only little evidence exists on the prognostic relevance of post-interventional changes of TR severity in patients after TMVR.

In the present study, we aimed (i) to investigate the prognostic impact of baseline concomitant TR on mid-term and long-term survival, (ii) to analyze frequency and severity of post-interventional TR after TMVR at 30-day and 1-year follow-ups and (iii) to detect potential implications caused by post-interventional changes of TR severity for long-term prognosis in a large prospective cohort.

## Methods

All patients treated for MR by transcatheter edge-to-edge repair at our institution between June 2010 and March 2018 were screened for inclusion. TMVR were all performed using MitraClip^®^-systems classic or NT before the introduction of the latest generation (NTR/XTR). Patients primarily undergoing a combination of MitraClip^®^-implantation with additionally simultaneous implantation of other forms of TMVR as well as patients with an unsuccessful index procedure were excluded. Additionally, all patients receiving interventional therapy for TR until the end of observational period (March 2018) were also excluded. All included subjects were adults (≥ 18 years) with moderate-to-severe or severe MR despite optimal medical treatment, including cardiac resynchronization therapy when indicated. Risk for surgery was assessed by an interdisciplinary Heart Team based on scoring systems (e.g., logistic Euroscore) and individual factors, such as frailty and comorbidities. Procedures were predominantly performed under general anesthesia. All patients were invited to undergo follow-up visits after 1 and 12 months for re-assessment of symptoms, echocardiographic evaluation and blood tests. Long-term survival or date of death, respectively, was retrieved from entries in our centers’ patient records or from an enquiry at the Rhineland-Palatinate bureau of vital statistics at March 8, 2018.

### Study endpoint

Primary outcome of this study was mortality of all causes. The secondary outcome comprised dyspnoea reduction classified by New York Heart Association (NYHA) class.

### Definitions

In accordance to the recommendations of the Mitral Valve Academic Research Consortium/MVARC [12]), technical success was defined as ability to deploy the device as intended and successful retrieval of the delivery system without peri-procedural mortality or need of emergency surgery or intervention. Due to the study design as a retrospective registry, device success and procedural success were adjusted from MVARC-recommendations to discharge conditions: device success at discharge was defined as successful placement of the device without procedural mortality or stroke, missing evidence of functional failure of the device or device-related complications until discharge and post-interventional reduction of MR to optimal or acceptable levels without significant mitral valve stenosis. The definition of procedural success at discharge included device success achieved in the absence of major clinical complications (according to MVARC [12]). MR and TR were graded by experienced echocardiographers according to society recommendations [13, 14]; TR severity was denominated in 4 grades: no/trace, mild, moderate, and severe (comprising the subgrades “severe”, “massive” and “torrential”, as defined by the most recent TR-classification scheme [15]). Renal insufficiency was defined by a glomerular filtration rate < 60 ml/min*kg. Pulmonary hypertension was determined by invasive measurements (if available at baseline) or echocardiographic high probability, according to guidelines [16]. For statistical evaluation of sPAP (systolic pulmonary arterial pressure), only values derived by non-invasive means (echocardiographic assessment of right ventricular systolic pressure derived from RV/RA-gradient plus estimated central venous pressure) were used. Obesity was defined as BMI ≥ 30 kg/m^2^. Echocardiographic left and right ventricular analyses and quantification were based on transthoracic echocardiography measurements in accordance to ASE/EACVI recommendations; RV dysfunction was predominantly defined by a reduced TAPSE (tricuspid annular plane systolic excursion) of < 17 mm [17].

### Statistical analysis

Continuous parameters are presented as median and interquartile range (IQR) when non-normally distributed (as tested by Kolmogorov–Smirnov and Shapiro–Wilk tests), and otherwise absolute numbers and percentages. Continuous variables were compared using the Wilcoxon–Whitney *U* test or Wilcoxon signed rank test and categorical variables with Fisher’s exact or chi^2^ test, as appropriate. We compared TMVR patients with severe vs. non-severe-grade TR as well as patients with post-interventional TR reduction to those without, including Kaplan–Meier Curves. Cox regression analyses were computed to examine the impact of TR at baseline as well as post-procedural changes of TR grade on short- and long-term mortality. Results were presented as Hazard Ratios (HR) with 95% confidence interval (CI) (i) univariate/unadjusted and (ii) multivariate/adjusted for factors which had been identified as having relevant impact on patients’ long-term prognosis in our cohort [18]: patients’ age at the time of procedure, gender, NYHA class before intervention, left ventricular ejection fraction (LVEF) at baseline, coronary artery disease, chronic obstructive pulmonary disease (COPD), renal function/baseline creatinine values, peri-interventional reduction of MR-grade, as well as existence of a pacemaker, in a multivariate fashion. A propensity match model was calculated for differences in the distribution of no/trace and mild vs. moderate and severe MR grades at discharge.

The software SPSS^®^ (IBM Corp. Released 2016. IBM SPSS Statistics for Windows, Version 24.0. Armonk, NY: IBM Corp.) was used for computerised analysis. *P* values of < 0.05 (two-sided) were considered to be statistically significant.

## Results

### Enrolment, baseline characteristics, survival and TR-assessment

**Fig. 1:**
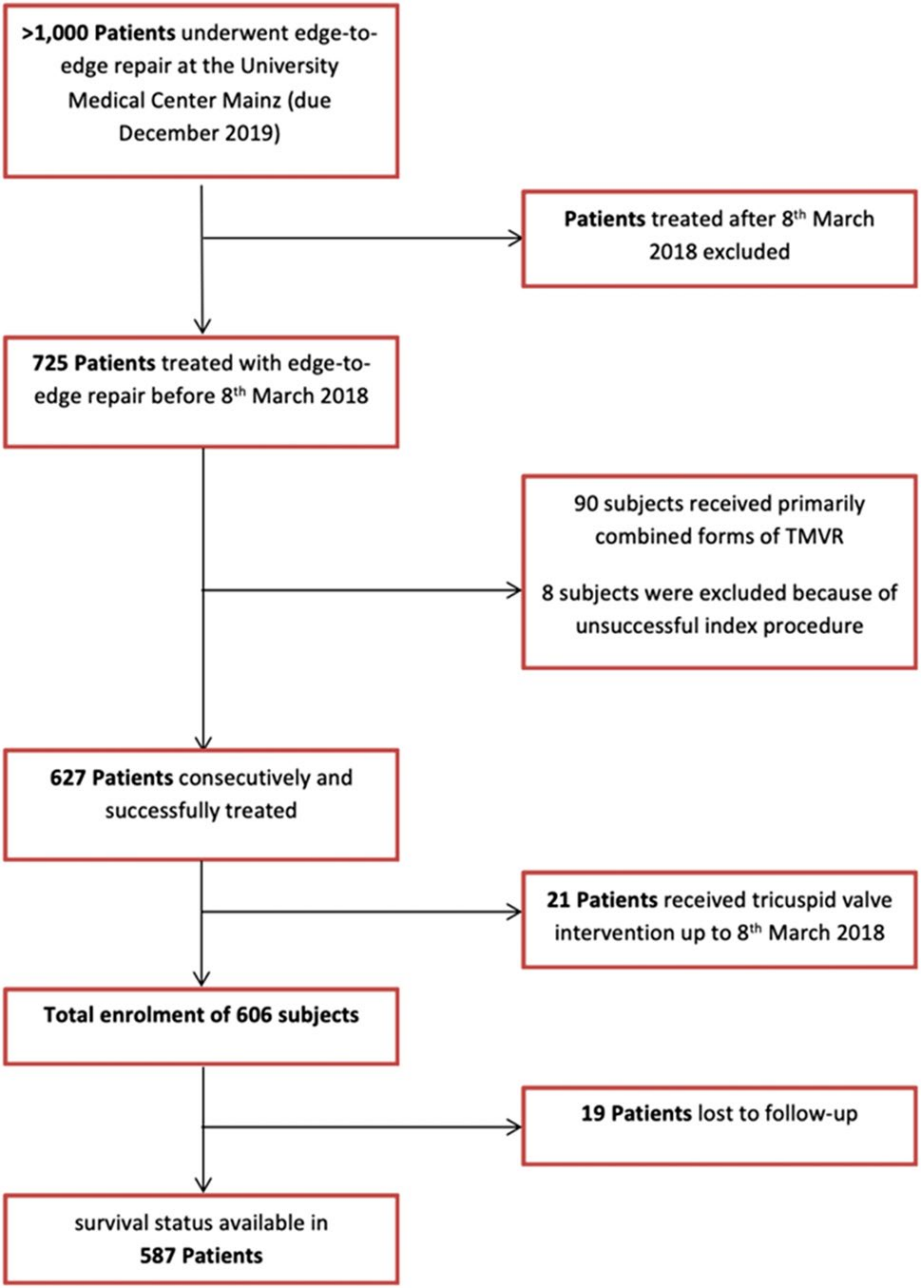
Flow diagram of patients’ enrolment for analysis

Between 09 June 2010 and 08 March 2018, 725 consecutive patients underwent percutaneous edge-to-edge-therapy at our center. Of those, 119 were excluded: 90 (12.4%) had primarily undergone a simultaneous combination of edge-to-edge repair and other forms of TMVR (e.g., interventional annuloplasty or chordal reconstruction), technical failure occurred in 8 patients (1.3%/technical success 98.7%; resulting mitral stenosis, leading to abortion of clip implantation in 6 patients; hemodynamic instability before insertion of the transseptal sheath in 1 patient, pericardial tamponade before introduction of the implantation with peri-interventional death in 1 patient), and 21 subjects (2.9%) had additionally undergone interventional tricuspid valve repair until the end of the follow-up period (mostly, due to severe symptomatic TR; TR repair was performed in various time intervals after TMVR). In total, 606 patients were included (Fig. 1**)**.

At the time of the index procedure, mean age was 78.5 ± 7.3 years [median 79.1 (IQR 74.1/83.7); 88.1% aged over 70 years] and sex distribution was nearly balanced (46.5% females). Leading etiology was FMR in 56.4%, DMR was present in 31.2% and mixed pathomechanism in 12.4%. The mean logistic Euroscore was 29.7% ± 17.0 [median 26.0 (18.4/38.7)]. Mean LVEF was moderately impaired [41.6% ± 13.4; median 42.0% (30.0/55.0)]. MR at baseline was classified severe in 91.9%, and moderate in 8.1% of the treated patients. Regarding dyspnoea, 99.4% had been symptomatic and 89.2% were graded as NYHA classes III/IV. Mean BNP level was elevated (961 ± 1116 pg/ml). Adverse events during the index visit were recorded in 8 subjects (1.3%:2/0.3% immediate surgical treatment during intervention, 2/0.3% peri-interventional myocardial infarction, 3/0.5% stroke, 2/0.3% hemodynamically relevant pericardial effusion). MR reduction was achieved in 94.0%. At discharge, device success was observed in 92.7% and procedural success in 91.7%.

**Fig. 2:**
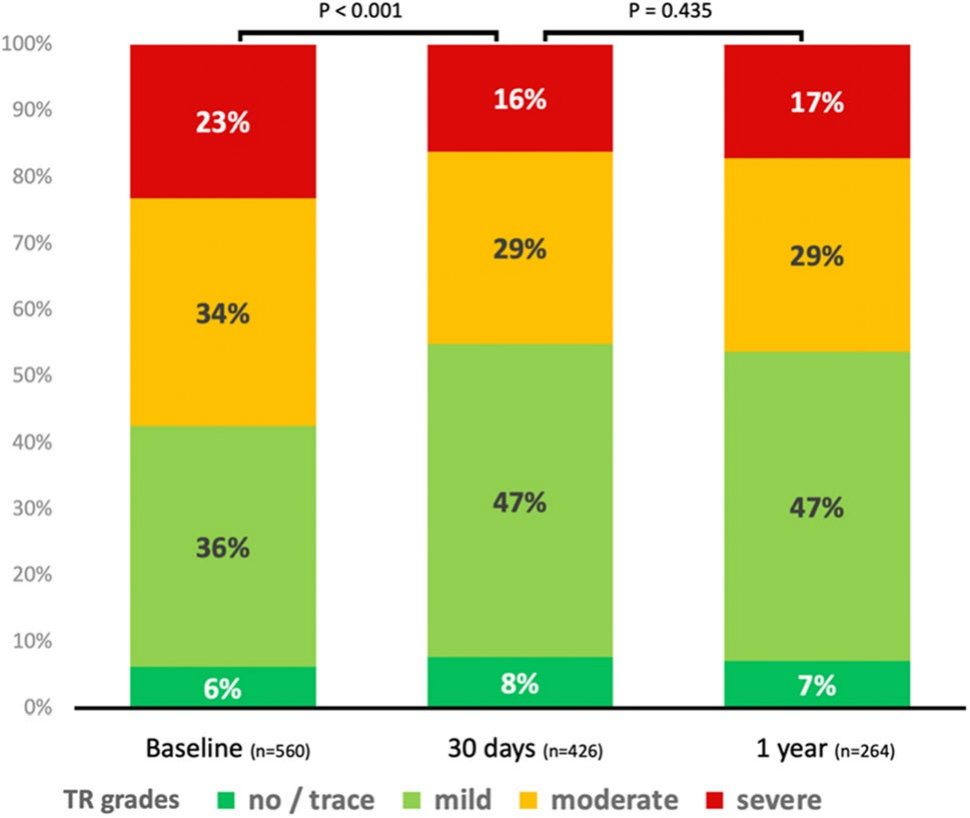
Changes in concomitant Tricuspid Valve regurgitation severity grades from baseline to 1-year follow-up. Post-interventional reduction of Tricuspid Regurgitation (TR): echocardiographic assessments at baseline, 30 days and 1 year

After a median follow-up of 511 [IQR161/981] days (mean 674 ± 619), survival status was available in 96.9%. Whereas in-hospital mortality was 2.5%, survival was 94.5% after one month, 74.5% after 1 year, 54.5% after 3 years, 37.6% after 5 years and 21.7% after 7 years. At baseline, echocardiographic assessment of TR was available in 92.4%; TR was categorized severe in 23.2%, medium in 34.3%, mild in 36.3% and no/trace in 6.3%. At 1-month [median time 43 (IQR37/55) days], echocardiographic examination results were available in 75.0% of the living patients (70.3% of the whole group) with TR graded as severe in 16.0%, medium in 29.1% and mild in 47.2%, whereas no/trace TR was found in 7.7% of the patients. At 1-year follow-up [median 366 (IQR350/377) days, echocardiography allowing TR quantification available in 63.2% of the surviving patients/42.1% of the whole group], 17.0% were graded as severe TR, 29.2% moderate, 46.6% mild and 7.2% no/trace. While changes in TR severity at both follow-ups versus baseline findings were significant (*p* < 0.001 at 30 days, *p* = 0.024 at 1 year), no significant differences were found regarding changes between 1-month and 12-month assessments (*p* = 0.435) (Fig. 2**)**.

For further statistical analyses regarding the impact of post-interventional TR reduction, only subjects with detectable (i.e., mild, medium or severe) TR at baseline (*n* = 525, 93.7% of the individuals with echocardiographic assessment), were included. At 1-month, improvement of at least 1 TR grade was documented in 34.9% (of  *n* = 378) of these patients; at 1 year, TR reduction compared to baseline was found in 35.3% (*of  n* = 235) of the patients.

### Differences in baseline characteristics between the groups

The groups of patients with severe vs. non-severe grade TR before intervention were comparable regarding most parameters, including gender, cardiovascular risk factors and etiology (Table 1**)**. In median, patients with severe TR were older [81.1 (76.6/84.9) vs. 78.5 (73.5/83.3) years, *p* < 0.001] and atrial fibrillation was more common (83.8 vs. 66.7%, *p* < 0.001) in patients with severe TR. While there was no difference regarding distribution of baseline MR grades, MR grades at discharge were lower in patients with severe TR (remaining moderate or severe MR in 20.7% vs. 30.5%, *p* = 0.030). Patients with severe TR presented with slightly higher Creatinine values at 30 days [1.39 (1.05/1.91) vs. 1.23 (0.99/1.60) mg/dl, *p* = 0.037].

**Table 1:** Patients’ characteristics stratified for severe vs. non-severe TR before TMVR

Parameter	Severe TR at baseline (*n* = 148, 25.6%)	TR grade 0–2 at baseline (*n* = 431, 74.4%)	*p *value
Age at procedure [years]	81.1 (76.6/84.9)	78.5 (73.6/83.3)	** < 0.001**
Age > 70 years	125 (96.2%)	371 (86.3%)	**0.001**
Female gender	71 (54.6%)	196 (45.6%)	0.072
Height [cm]	167 (162/172)	169 (162/175)	0.059
Weight [kg]	70 (65.0/80.0)	72.5 (65.0/84.0)	0.229
BMI [kg/m^2^]	25.5 (23.6/27.7)	25.6 (23.1/28.0)	0.980
NYHA III or IV (baseline)	106 (93.0%)	342 (89.3%%)	0.287
Cardiovascular risk factors			
Obesity	14 (10.9%)	65 (15.4%)	0.260
Art. Hypertension	114 (87.7%)	366 (85.1%)	0.567
Diabetes mellitus	32 (24.6%)	123 (28.6%)	0.434
Intervention parameters			
FMR	76 (58.5%)	241 (56.0%)	0.686
DMR	41 (31.5%)	134 (31.2%)	1.000
Mixed etiology	13 (10.1%)	55 (12.8%)	0.447
Logistic Euroscore I [%]	27.5 (19.0/40.0)	26.0 (18.1/37.5)	0.350
Comorbidities			
COPD	14 (10.8%)	65 (14.9%)	0.311
PAH	71 (54.6%)	254 (59.2%)	0.363
Atrial fibrillation	109 (83.8%)	287 (66.7%)	** < 0.001**
Renal insuffiency	72 (55.4%)	200 (46.6%)	0.089
CAD	81 (52.3%)	279 (65.0%)	0.602
History of myocardial infarction	26 (20.0%)	236 (29.3%)	**0.043**
PAD	24 (10.0%)	44 (10.2%)	1.000
History of stroke	19 (14.6%)	46 (10.7%)	0.216
History of cardiac surgery	29 (22.3%)	107 (24.9%)	0.641
History of surgical MVR/r	2 (1.5%)	7 (1.6%)	1.000
Pacemaker	42 (32.3%)	123 (28.9%)	0.443
Implantable cardioverter-defibrillator	17 (13.1%)	67 (15.6%)	0.575
Medication			
Diuretics	125 (96.9%)	394 (91.8%)	0.050
RAS-Blockers	100 (77.5%)	363 (84.6%)	0.063
Betablockers	105 (81.4%)	354 (82.5%)	0.793
Echocardiography			
LVEF [%] baseline/30 days/1 year	47 (30/55)	40 (30/55)	0.073
	50 (35/55)	42 (30/55)	**0.030**
	46 (39/55)	45 (30/55)	0.581
MR (grade)* baseline	0: 0.0%	0: 0.0%	0.683
	1: 0.0%	1: 0.0%	
	2: 7.7%	2: 2.2%	
	3: 92.3%	3: 97.8%	
MR (grade)* discharge	0: 9.5% 1: 69.8% 2: 18.3% 3: 2.4%	0: 7.2% 1: 62.4% 2: 26.4% 3: 4.1%	**0.030**
MR (grade)* 30 days	0: 4.5% 1: 50.0% 2: 39.8% 3: 5.7%	0:8.3% 1:48.6% 2: 36.7% 4: 6.4%	0.550
MR (grade)* 1 year	0: 3.8% 1: 46.2% 2: 42.3% 3: 7.7%	0: 4.4% 1: 52.0% 2: 36.8% 3: 6.9%	0.435
TR (grade)* 30 days	0: 1.2% 1: 22.1% 2: 31.4% 3: 45.3%	0: 8.8% 1: 54.2% 2: 28.8% 3: 8.2%	** < 0.001**
P_mean_ MV [mmHg] baseline/30 days	3.0 (2.0/4.0) 3.0 (2.0/4.0)	2.0 (1.9/3.6) 3.0 (2.4/5.0)	0.356 0.050
RV dysfunction baseline/30 days	56.3% (76)	30.7% (122)	** < 0.001**
	45.9% (39)	34.9% (111)	0.077
sPAP [mmHg] baseline/30 days	53.0 (43.5/64.0) 45.0 (39.0/53.3)	50.0 (45.0/60.0) 46.0 (40.0/54.0)	0.278 0.392
TAPSE [cm] baseline/30 days	1.6 (1.4/2.1) 1.8 (1.4/2.0)	1.7 (1.4/2.1) 1.7 (1.5/2.1)	0.316 0.569
Laboratory examinations			
Creatinine [mg/dl] baseline/30 days	1.31 (1.00/1.81)	1.23 (0.93/1.69)	0.097
	1.39 (1.06/1.91)	1.23 (0.99/1.60)	**0.037**
BNP [pg/ml] baseline/30 days	678 (435/1530) 478 (290/994)	545 (245/1142) 481 (257/880)	0.061 0.304
hsTnI [pg/ml] baseline/30 days	19.1 (8.0/47.4) 13.7 (7.1/27.2)	18.9 (7.4/46.7) 12.4 (5.4/27.7)	0.846 0.494
Exercise testing			
6 min Walk-test [m/6 min] baseline/30 days	152 (35/250) 311 (194/355)	250 (127/351) 300 (225/395)	0.108 0.315

*BMI* body mass index, *NYHA* New York Heart Association, *FMR* functional mitral valve regurgitation, *DMR* degenerative mitral valve regurgitation, *COPD* chronic obstructive pulmonary disease, *PAH* pulmonary artery hypertension, *CAD* coronary artery disease, *PAD* peripheral artery disease, *MVR* mitral valve replacement, *RAS* Renin-angiotensin, *LVEF* left ventricular ejection fraction, *MR/TR* mitral/tricuspid valve regurgitation, *P*_*mean*_ mean pressure, *MV* mitral valve, *RV* right ventricular, *sPAP* systolic pulmonary artery pressure, *TAPSE* tricuspid annular plane systolic excursion, *BNP* brain natriuretic peptide, *hsTnI* high sensitive troponin I

*Classified in 4 grades: 0 = no/trace, 1 = mild, 2 = moderate or moderate-severe, 3 = severe

Bold values indicates p-values <0.05

When comparing groups with post-interventional TR reduction to those without, both groups were nearly balanced regarding most baseline parameters (Table 2**)**. As expected, higher baseline grades of TR were more often found in patients with a post-interventional decrease in TR severity (moderate or severe TR in 90.9 vs. 35.2%, *p* < 0.001). Furthermore, moderate or severe MR at discharge was less common in patients with TR reduction (17.7 vs. 33.8%, *p* = 0.002). Logistic Euroscore I was predominantly indicating high surgical risk in both groups, yet with a lower median value for patients with post-interventional TR reduction [22.0% (16.1/30.5) vs. 26.5% (18.5/37.9), *p* = 0.015]. Regarding medical therapy, the frequency of intake of RAS-blockers was higher (88.6 vs. 79.7%, *p* = 0.032) in patients with TR-grade reduction.

**Table 2:** Patients’ characteristics (only mild, medium or severe TR-grade at baseline) stratified for post-interventional reduction of baseline TR one month after TMVR

Parameter	TR grade reduced (*n* = 132, 34.9%)	TR grade unchanged (*n* = 246, 65.1%)	*p* value
Age at procedure [years]	81.1 (76.6/84.9)	78.5 (73.6/83.3)	0.724
Age > 70 years	117 (88.6%)	221 (89.8%)	0.728
Female gender	58 (43.9%)	114 (46.3%)	0.666
Height [cm]	169 (162/174)	168 (162/175)	0.932
Weight [kg]	73 (65.0/83.0)	72.0 (65.0/83.0)	0.657
BMI [kg/m^2^]	25.7 (23.4/27.7)	25.3 (22.9/27.8)	0.586
NYHA III or IV (baseline)	111 (91.0%)	199 (88.4%%)	0.585
Cardiovascular risk factors			
Obesity	19 (14.5%)	35 (14.3%)	1.000
Art. Hypertension	114 (86.4%)	207 (84.1%)	0.652
Diabetes mellitus	34 (25.8%)	64 (26.0%)	1.000
Intervention parameters			
FMR	71 (53.8%)	1143 (58.1%)	0.447
DMR	47 (35.6%)	71 (28.9%)	1.000
Mixed etiology	14 (10.6%)	32 (13.0%)	0.621
Logistic Euroscore I [%]	22.0 (16.1/30.5)	26.5 (18.5/37.9)	**0.015**
Comorbidities			
COPD	20 (15.2%)	33 (13.4%)	0.644
PAH	82 (62.1%)	145 (58.9%)	0.583
Atrial fibrillation	100 (75.8%)	171 (69.5%)	0.231
Renal insuffiency	50 (37.9%)	117 (47.8%)	0.082
CAD	76 (57.6%)	160 (65.0%)	0.181
History of myocardial infarction	31 (23.5%)	68 (27.6%)	0.043
PAD	9 (6.8%)	24 (9.8%)	0.445
History of stroke	17 (12.9%)	29 (11.8%)	0.744
History of cardiac surgery	24 (18.2%)	56 (22.8%)	0.355
History of surgical MVR/r	2 (1.5%)	3 (1.2%)	1.000
Pacemaker	39 (29.5%)	75 (30.5%)	0.907
Implantable cardioverter-defibrillator	20 (15.2%)	40 (16.3%)	0.883
Medication			
Diuretics	123 (93.2%)	227 (92.3%)	0.839
RAS-Blockers	117 (88.6%)	196 (79.7%)	**0.032**
Betablockers	109 (82.6%)	207 (84.1%)	0.771
Echocardiography			
LVEF [%] baseline /30 days/1 year	45 (35/55) 50 (35/55) 45 (40/55)	41 (30/55) 42 (30/55) 40 (30/55)	0.142 **0.011** 0.272
MR (grade)* baseline	0: 0.0% 1: 0.0% 2: 5.3% 3: 94.7%	0: 0.0% 1: 0.0% 2: 7.7% 3: 92.3%	0.376
MR (grade)* discharge	0: 6.2% 1: 76.2% 2: 15.4% 3: 2.3%	0: 7.4% 1: 58.7% 2: 29.7% 3: 4.1%	**0.002**
MR (grade)* 30 days	0: 8.3% 1: 59.8% 2: 30.3% 3: 1.5%	0: 7.3% 1: 44.0% 2: 40.7% 3: 8.1%	**0.001**
MR (grade)* 1 year	0: 0.0% 1: 56.8% 2: 30.9% 3: 12.3%	0: 5.9% 1: 48.0% 2: 42.1% 3: 3.9%	0.530
TR (grade)* baseline	1: 9.1% 2: 55.3% 3: 35.6%	1: 54.9% 2: 29.3% 3: 15.9%	** < 0.001**
TR (grade)* 30 days	0: 12.9% 1: 66.7% 2: 20.5% 3: 0.0%	0: 4.4% 1: 38.1% 2: 33.7% 3: 23.8%	** < 0.001**
P_mean_ MV [mmHg] baseline/30 days	3.0 (2.0/4.0) 3.0 (2.0/4.0)	2.0 (1.0/3.1) 3.0 (2.5/5.0)	0.105 **0.048**
RV dysfunction baseline/30 days	35.0% (43) 30.5% (40)	36.9% (86) 40.8% (109)	0.716 **0.048**
sPAP [mmHg] baseline/30 days	54 (47/60) 45 (39/52)	50 (43/60) 47 (40/55)	**0.035** 0.205
TAPSE [cm] baseline/30 days	1.8 (1.4/2.2) 1.8 (1.5/2.1)	1.6 (1.3/1.9) 1.7 (1.4/2.1)	**0.016** 0.787
Laboratory examinations			
Creatinine [mg/dl] baseline/30 days	1.20 (0.97/1.67) 1.21 (0.99/1.66)	1.21 (0.93/1.68) 1.25 (1.00/1.66)	0.885 0.524
BNP [pg/ml] baseline/30 days	443 (238/965) 417 (236/646)	594 (253/1470) 514 (278/1007)	0.068 **0.007**
hsTnI [pg/ml] baseline/30 days	14.3 (6.1/41.8) 13.3 (6.2/24.4)	17.9 (6.6/40.0) 11.9 (5.4/28.0)	0.621 0.540
Exercise testing			
6 min Walk-test [m/6 min] baseline/30 days	400 (288/420) 316 (216/368)	155 (0/390) 300 (238/367)	**0.040** 0.347

*BMI *body mass index, *NYHA* New York Heart Association, *FMR* functional mitral valve regurgitation, *DMR *degenerative mitral valve regurgitation, *COPD *chronic obstructive pulmonary disease, *PAH *pulmonary artery hypertension, *CAD *coronary artery disease, *PAD* peripheral artery disease, *MVR *mitral valve replacement, *RAS* Renin-angiotensin, *LVEF* left ventricular ejection fraction, *MR/TR* mitral/tricuspid valve regurgitation, *P*_*mean*_ mean pressure, *MV* mitral valve, *RV* right ventricular, *sPAP* systolic pulmonary artery pressure, *TAPSE* tricuspid annular plane systolic excursion, *BNP* brain natriuretic peptide, *hsTnI* high sensitive troponin I

*Classified in 4 grades: 0 = no/trace, 1 = mild, 2 = moderate, 3 = severe

Bold values indicates p-values <0.05

### Prognostic impact of baseline TR and its secondary reduction regarding survival

**Fig. 3:**
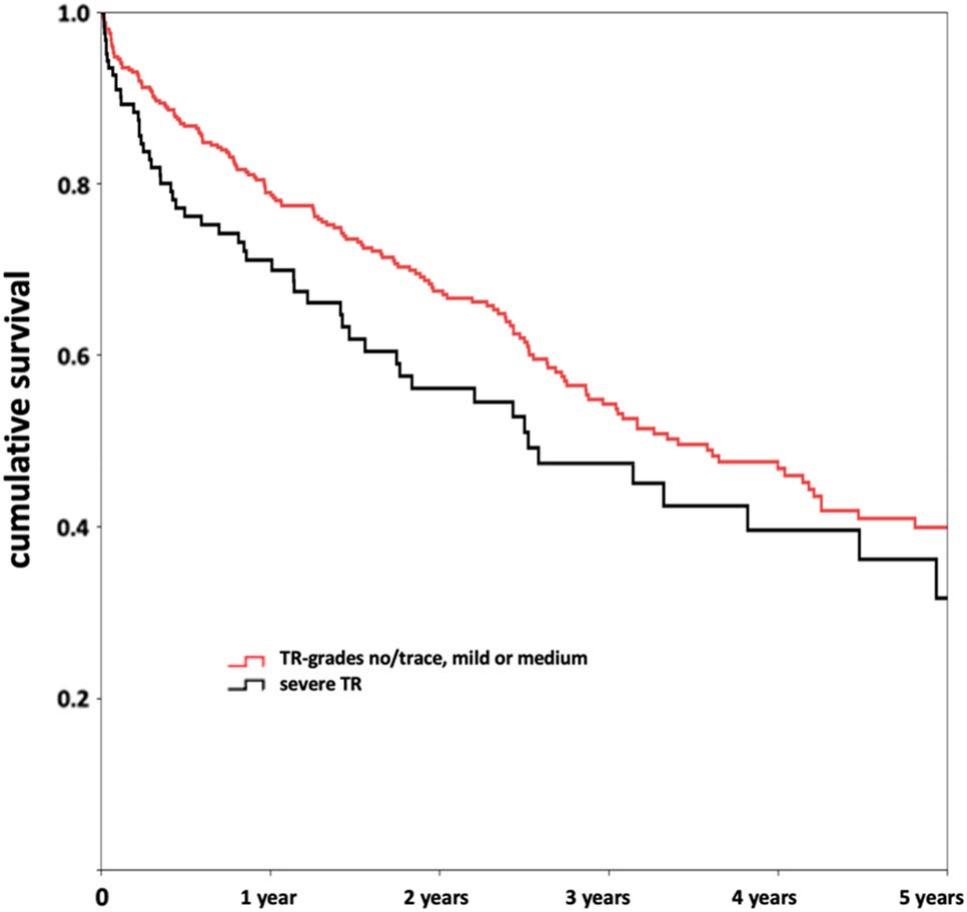
Kaplan–Meier Curves for long-term survival after TMVR, stratified for baseline Tricuspid Valve regurgitation severity. Cumulative survival after TMVR by edge-to-edge repair is dependent on baseline Tricuspid Regurgitation (TR) severity

With regard to long-term survival of TMVR patients with severe TR in comparison to those with lower grades of TR at baseline, relevant differences were found (Fig. 3). While in-hospital (3.8% vs. 2.3%, *p* = 0.356) and 30-day mortality (7.6 vs. 5.3%, *p* = 0.370) were similar, 1-year survival was significantly reduced in patients with severe TR (65.2% vs. 77.0%, *p* = 0.030) with still relevant differences at 3 (45.8% vs. 56.7%, *p* = 0.193) and at 5 years (25.0% vs. 41.1%, *p* = 0.224). This finding was even more pronounced in FMR patients (1-year survival 56.9% vs.77.0%, *p* = 0.007), in contrast to DMR patients (1-year survival 80.6% vs. 78.7%, *p* = 1.000). Thus, severe baseline TR was identified as predictor for a higher 1-year mortality in the total cohort [crude HR 1.68 (95% CI 1.12–2.54), *p* = 0.013], even after adjustment for other factors influencing the long-term survival [adjusted HR 1.65 (95% CI 1.01–2.68), *p* = 0.044; HR after propensity matching for MR-grade at discharge 1.71 (95% CI 1.03–2.86), *p* = 0.040]. Regarding long-term survival until ultimate follow-up, HR for severe TR indicated a trend towards impaired survival [crude HR 1.34 (95% CI 0.99–1.81), *p* = 0.063, adjusted HR 1.36 (95% CI 0.96–1.92), *p* = 0.085]. For FMR patients, severe TR was predictive for impaired 1-year survival [crude HR 2.31 (95% CI 1.34–3.72), *p* = 0.002 as well as adj. HR 1.97 (95% CI 1.06–3.64), *p* = 0.031; HR after propensity matching for MR-grade at discharge 2.54 (95% CI 1.31–4.91), *p* = 0.006] with a borderline significance for the total follow-up period [crude HR 1.46 (95% CI 1.00–2.15), *p* = 0.052 as well as adj. HR 1.57 (95% CI 1.00–2.45), *p* = 0.049; HR after propensity matching for MR grade at discharge 1.63 (95% CI 1.00–2.66), *p* = 0.050].

**Fig. 4:**
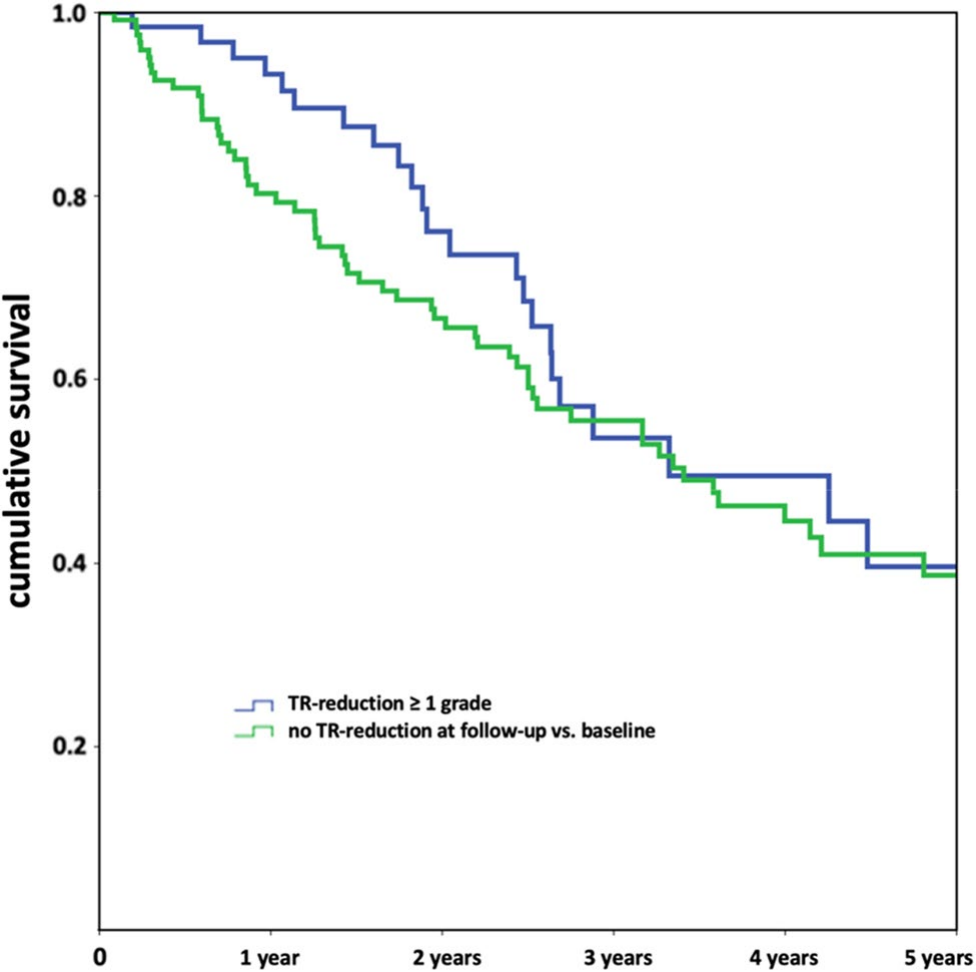
Kaplan–Meier Curves for long-term survival after TMVR, stratified for post-interventional change in Tricuspid Valve regurgitation grade (as assessed at 30-days Follow-up) compared to cumulative survival after TMVR by edge-to-edge repair is influenced by post-interventional reduction of concomitant Tricuspid Regurgitation (TR) severity

Patients with post-interventional TR reduction had a better long-term survival in comparison to those without: 88.5% vs. 81.0% at 1-year (*p* = 0.102) with persisting non-significant differences for later follow-ups. When focusing on FMR-patients, prognosis was significantly better in subjects with post-interventional TR reduction at 1-year follow-up (92.9% vs. 78.3%, *p* = 0.025), while later differences were still recognizable, but not of statistical significance (Fig. 4). For the whole cohort, HR indicated for increased mortality at 1 year for in patients lacking post-interventional TR reduction when adjusted for risk factors [crude HR 1.71 (95% CI 0.89–3.29), *p* = 0.107, adj. HR 2.18 (95% CI 1.02–4.68), *p* = 0.046, HR after propensity matching for MR grade at discharge 2.39 (95% CI 1.03–5.58), *p* = 0.043]. In FMR patients, missing post-interventional TR reduction could be identified as predictor for significantly impaired survival at 1 year [HR 3.31 (95% CI 1.15–9.58), *p* = 0.027, adj. HR 6.82 (95% CI 1.88–24.71), *p* = 0.003, HR after propensity matching for MR-grade at discharge 6.31 (95% CI 1.46–27.32), *p* = 0.014] while long-term HR was not significant [adj. HR 1.27 (95% CI 0.74–2.18), *p* = 0.381]. Reasons why the benefit in survival by TR reduction beyond one year is gradually losing significance, e. g. being be caused by statistical effects (e. g. due to the smaller numbers at risk) or the survival difference decreases gradually, remains subject to future studies.

### Post-interventional changes in right ventricular echocardiographic parameters

Patients with post-procedural TR reduction had a better baseline systolic right ventricular function as assessed by TAPSE [tricuspid annular plane systolic excursion, 1.8 (1.4/2.2) vs. 1.6 (1.3/1.9) cm, *p* = 0.016]. Post-procedural changes in TAPSE compared to baseline were not significant, neither for the whole cohort at one month (*p* = 0.069) and 1 year (*p* = 0.920), nor for the subgroup of patients presenting with reduction of TR severity at follow-up (*p* = 0.856 at 30 days, *p* = 0.475 at 1 year). When comparing the severe vs. non-severe baseline TR, no significant differences were observed in baseline TAPSE [1.6 (1.4/2.1) vs. 1.7 (1.4/2.1) cm, *p* = 0.316]. Furthermore, no relevant changes in TAPSE were found at 1 month (*p* = 0.151) and 12 months (*p* = 0.934) to baseline, even for the subgroup of patients with severe TR (*p* = 0.513 at 30 days, *p* = 0.228 at 1 year).

Systolic pulmonary arterial pressure (sPAP), as assessed by echocardiography, was significantly reduced by the procedure in the whole group, starting from 51 [45/60] mmHg reduced to 46 [39/54] mmHg at 30-day follow-up (*p* < 0.001) and 46 [0/53] mmHg at 1 year (*p* < 0.001 compared to baseline). Whereas baseline differences for the subgroups of severe vs. non-severe TR were not significant, patients with later TR reduction had higher assessed sPAP values before the procedure [53 (47/60) vs. 50 (43/60) mmHg, *p* = 0.035].

### TR and symptomatic benefit

New York Heart Association class reduction by at least one grade at one-month follow-up was found nearly equally distributed in groups with or without severe baseline TR (70.8 vs. 69.9%, *p* = 0.887). In contrast, patients with post-interventional TR decrease were significantly overrepresented in the group of patients reporting dyspnoea reduction (78.6 vs. 65.9%, *p* = 0.021). BNP values decreased in the whole cohort [median 588 (275/1283) at baseline vs. 503 (272/955) pg/ml at 30 days, *p* = 0.008], accordingly. Absolute BNP levels at 1 month were lower in the group with TR reduction [417 (236/646) vs. 515 (278/1007) pg/ml, *p* = 0.007]. Yet, the finding was not accompanied by a higher proportion of patients with declining BNP values [54.4% vs. 53.1%, *p* = 0.907], or significant differences in relative changes in BNP levels (in mean −  11.1 vs. − 28.3%, *p* = 0.214) between baseline and 30-day follow-ups.

## Discussion

In patients presenting with mitral valve disease, concomitant TR is a common finding. In our cohort, only 6% of the TMVR patients presented without detectable TR at baseline, and remarkably, 58% had a moderate or severe TR. One month after TMVR, this percentage exhibited a sustained decline to 35% without significant further changes after 1 year. Thus, and due to a higher follow-up rate at one month, TR reduction at 30 days was used for the evaluation of a prognostic and symptomatic impact. In 35% of the patients, concomitant TR was reduced. Recently, a large registry comprising more than 5, 000 DMR patients demonstrated that severity of concomitant TR is a relevant inverse predictor of long-term survival of these patients regardless of a medical or surgical management strategy regarding MR [8]. While symptomatic and prognostic benefits have been recently proven for edge-to-edge TMVR for FMR in a multi-centric prospective trial in the US [19], the discussion on optimal patient selection is still on-going, since another prospective trial with similar design did not confirm these results [20]. Several registries have investigated factors influencing the short- and mid-term survival; yet, data on long-term prognosis after TMVR are still limited. The German transcatheter mitral valve interventions registry (TRAMI) with more than 700 patients is the largest European multicenter cohort of patients treated with edge-to-edge TMVR therapy for MR; this registry was used to identify non-cardiac and cardiac comorbidities, which were relevant determinants of survival reporting a follow-up period of up to four years [21, 22]. Other registries, as the US TVT-Registry [23], the Transcatheter Valve Treatment Sentinel Pilot Registry [24], the ACCESS-EU Study [25] and the Italian GRASP-IT [26] registry with a reported follow-up of five years as well as recently published data from our patient cohort [18] mainly confirmed these findings.

Only a few studies have investigated the prognostic role of concomitant tricuspid valve disease in MR in the context of TMVR. In our cohort, severe vs. non-severe TR at baseline proved to be a relevant and severe TR was demonstrated to be an independent risk factor for survival. This finding is in accordance with the results of subgroup analyses from the TRAMI and GRASP-IT registry indicating that baseline TR severity has an independent and inverse impact on survival at 1 year after edge-to-edge therapy [27, 28]. A meta-analysis of 1,328 TAVI and 1, 001 MitraClip^®^ patients concluded that moderate-to-severe TR was a relevant determinant for increased mortality in left ventricular valvular heart disease [29]. Whether observed differences in survival between FMR and DMR patients in our study are subject to statistical bias, published data on surgically or medically treated DMR patients concluded that concomitant TR could also be a relevant determinant of survival in these patients [8] —or are caused by the completely different pathophysiology of MR etiologies as well as an impact of post-interventional changes in TMVR patients, has to be addressed by future analyses.

Confirming earlier published data, we found that TMVR is capable of influencing severity of concomitant TR. A reduction could be observed in 35% of our patients’ cohort, which is comparable to findings from other registries [10, 11]. Regarding the prognostic impact of peri-interventional development of TR severity, retrospective studies on patients undergoing transcatheter mitral valve edge-to-edge repair reported that also post-interventional severity of TR could account for impaired long-term prognosis [11, 30]. A mid-sized retrospective study on 139 patients with echocardiographic 30-day follow-up after interventional edge-to-edge therapy was the first indicating that a lack of post-interventional TR reduction is associated with a higher mortality in patients with significant TR at baseline [31]. In our present analysis, we confirmed that post-interventional reduction of TR severity evaluated at 1 month is sustainable nearly unchanged to one-year follow-up and may serve as an independent predictor for a significant prognostic benefit for patients undergoing TMVR by edge-to-edge repair over a long-term follow-up period, especially in the subgroup of FMR patients. Furthermore, we could document that reduction of TR severity might also be a surrogate for an increased incidence of symptomatic improvement in these patients.

Nevertheless, we found a relevant proportion of patients (75.1% of all patients with measurable TR and still 47.2% of patients with severe or medium baseline TR) not amenable to a post-interventional decrease in TR severity. With respect to the fact that TR remains untreated up till now in a majority of patients [32] and many MR patients are at elevated surgical risk, the possible need for interventional therapy for TR has gained much scientific attention over the last years. Several devices dedicated to transcatheter tricuspid valve repair have become available and promising results have been published in recent trials [33, 34]. Recently, a retrospective analysis on 106 patients from the TRAMI and 122 patients from the TriValve (Transcatheter Tricuspid Valve Therapies) with all severe MR and TR at baseline indicated an improved 1-year survival after additional tricuspid valve repair (TriValve) over isolated TMVR (TRAMI) [35]. Our results generate further evidence, that TMVR is capable of improving concomitant TR presumably by pressure and volume unloading with secondary right ventricular remodeling representing an independent predictor for a favorable prognosis. Although an actual benefit of a “staged approach” remains theoretical and thus subject to future prospective studies, results of our study might point to the notion that additional tricuspid valve repair might be advantageous in patients with residual relevant TR after TMVR.

### Limitations

Due to the retrospective observational monocentric design on an all-comer population of consecutive patients undergoing TMVR lacking a control group, a potential selection bias cannot be excluded and should be taken into account. While follow-up regarding survival status is nearly complete, echocardiographic assessment of TR was available in 92.4% at baseline, in 75% at 1-month follow-up and in 63% at 1 year in this retrospective real-world cohort. Although most baseline parameters with individual impact on survival were distributed equally and also multivariate analyses pointed to a better survival of patients with post-interventional TR reduction, a slightly but significantly different Euroscore of both groups could have influenced results. Furthermore, a significantly higher mean age, prevalence of atrial fibrillation and RV dysfunction as well as non-significant differences in renal function and baseline medication should be taken into account as potential competing risks for the observed higher mortality of patients with severe baseline TR. According to MVARC recommendations, and due to the study design, all-cause mortality was defined as primary endpoint without further sub-stratification [12]. Regarding accuracy of the parameters presented, grading of TR was performed by experienced echocardiographers from our center in a semi-quantitative way according to current guidelines [13–15] but not confirmed by an external core laboratory. sPAP values as provided were not taken from invasive measurement, but solely estimated by non-invasive means, thus, etiology of PH (pre- vs. post-capillary) could not be discriminated. Furthermore, TR severity could also be influenced by volume load (regredient BNP values at follow-ups).

## Conclusion

In a large retrospective monocentric analysis, we could generate robust evidence that not only concomitant TR at baseline, but also lacking peri-interventional improvement of TR are both relevant and independent risk factors for adverse long-term prognosis after TMVR by edge-to-edge repair. More than a third of patients with baseline TR are amendable to secondary and persistent TR reduction by the procedure and these proved to be prone to a more favorable symptomatic and prognostic outcome. As still a relevant proportion of patients does not achieve a direct peri-interventional TR reduction and the possibilities and indications for transcatheter tricuspid valve therapy are steadily growing, future studies are needed on a potential prognostic impact of additional tricuspid valve repair in these patient groups.

## Abbreviations

ASE: American Society of Echocardiography
CI: Confidence interval
EACVI: European Association of Cardiovascular Imaging
FDA: (US) Food and Drug Administration
IQR: Interquartile range
LVEF: Left ventricular ejection fraction
MR: Mitral valve regurgitation (DMR: degenerative, FMR: functional)
MVARC: Mitral Valve Academic Research Consortium
OR: Odds ratio
TMVR: Transcatheter mitral valve repair
TR: Tricuspid valve regurgitation
